# Architectures, Protocols and Algorithms of Sensor Networks—Second Edition

**DOI:** 10.3390/s26072197

**Published:** 2026-04-02

**Authors:** Bartłomiej Płaczek

**Affiliations:** Institute of Computer Science, University of Silesia, Będzińska 39, 41-200 Sosnowiec, Poland; bartlomiej.placzek@us.edu.pl

## 1. Introduction

Sensor networks have become a background technology for a wide range of applications in industrial automation, smart cities, intelligent transportation systems, healthcare monitoring, environmental sensing, precision agriculture, and supervision of critical infrastructure [1,2,3,4]. By enabling continuous data acquisition, processing, and transmission across distributed environments, sensor networks effectively support advanced intelligent services. However, the increasing scale, heterogeneity, and performance demands of these systems impose significant constraints on network architectures, communication protocols, and the design of specialized algorithms tailored to them. We are witnessing the evolution of sensor networks from relatively simple data-collection solutions to complex, distributed cyber-physical systems. For real-world implementations, such systems must support long-term autonomous operation while ensuring reliability, scalability, and low energy consumption [5,6,7]. These requirements have motivated active research across many areas, including the proliferation of tiny, resource-constrained devices, heterogeneous communication technologies, application-specific quality-of-service techniques, low-latency transmission in industrial control, and high reliability, e.g., in healthcare monitoring [8,9]. Addressing the above-mentioned challenges requires innovative solutions related to different layers of the system, including hardware, network architectures, medium access protocols, intelligent data processing, and adaptive resource management algorithms.

One should also note that sensor networks play a central role in the Internet of Things (IoT), serving as the indispensable interface between the physical and digital worlds [10,11,12]. IoT systems rely on sensor networks and embedded devices to capture real-time information about various processes, human activities, and environmental conditions. The effectiveness of IoT applications in smart manufacturing, logistics, telemedicine, ecological sustainability, and other domains directly depends on the robustness, efficiency, and security of the sensor networks that provide input data. Therefore, advancements in sensor network design are essential for developing scalable IoT systems that can support the connectivity of numerous devices, distributed intelligence at the edge, and seamless integration with cloud and multi-access edge computing platforms. Simultaneously, the implementation of IoT systems in new scenarios introduces novel challenges for sensor networks. These challenges include the need for deterministic communication in industrial environments, energy-aware operation in large-scale deployments, support for embedded machine learning, and resilience against evolving security threats. Consequently, the convergence of sensor networks with edge computing, artificial intelligence, and next-generation communication technologies has become a central focus of research [13,14,15]. This convergence necessitates the development of innovative architectures that accommodate heterogeneous devices, protocols that adapt to dynamic network conditions, and algorithms that optimize key performance metrics such as energy consumption, latency, throughput, and network lifetime.

This Special Issue, titled “Architectures, Protocols and Algorithms of Sensor Networks—Second Edition”, builds upon the foundations established in the first edition and reflects the current state of the art in sensor network research. The eleven contributions included in this Special Issue address complementary aspects of sensor network design and operation, encompassing industrial communication architectures, real-world deployment studies, analytical performance modeling, learning-driven optimization techniques, energy-efficient algorithms, secure embedded platforms, and post-quantum cryptographic solutions. By combining theoretical analysis with experimental validation and practical implementation, these contributions collectively provide valuable insights into the challenges and opportunities shaping new solutions in sensor networks and their role within the broader IoT landscape.

## 2. Overview of Contributions

The contributions in this Special Issue span multiple layers of the system stack, encompassing Internet-scale measurement and distributed coordination, communication protocols and network architectures, analytical performance modeling, learning-driven optimization techniques, and hardware–software platforms that address energy efficiency, reliability, and security. Taken together, these works illustrate how advances in theory, algorithms, system design, and experimental validation support the development of scalable, intelligent, and resilient sensor network solutions for diverse application domains. The individual contributions are briefly described in the following subsections.

### 2.1. Architectures and Protocols for Industrial Sensor Networks

In the paper “Two-Phase Distributed Genetic-Based Algorithm for Time-Aware Shaper Scheduling in Industrial Sensor Networks” (Contribution 2), the authors address a challenge in Time-Sensitive Networking (TSN) for industrial sensor networks, namely the scalability and deployability limitations of centralized Time-Aware Shaper (TAS) scheduling under the IEEE 802.1Qbv standard. The authors observe that existing global schedulers often produce fragmented Gate Control Lists (GCLs) that exceed the per-port entry limits of commercial switches, thus hindering practical deployment in large-scale networks.

To overcome these limitations, the authors propose a two-phase distributed genetic-based algorithm (2PDGA). The first phase performs a network-wide genetic optimization to determine routing paths and transmission offsets, producing a conflict-free baseline schedule. The second phase refines the schedule locally at each switch, merging time windows and enforcing hardware-specific GCL constraints through lightweight coordination. Extensive evaluations across multiple topologies, network sizes, and guard-band configurations demonstrate that the proposed approach achieves high compliance rates under strict hardware constraints, maintains low latency, and improves the feasibility of deploying large-scale TSN-enabled industrial sensor networks.

In Contribution 7, Lacasa et al. focus on architectural challenges associated with deploying Industrial Internet of Things solutions in operational factory environments. Within the Industrializable Industrial Internet of Things (I3oT) paradigm, the authors emphasize the importance of reusing existing industrial infrastructures, such as programmable logic controllers (PLCs) and IT/OT networks, to minimize deployment costs and operational disruptions.

The proposed Cross-PLC platform enables passive and vendor-agnostic data extraction from heterogeneous PLCs by acting as a virtual PLC that aggregates and standardizes information for I3oT applications. Supporting both native PLC communication and HTTP/JSON-based interfaces, Cross-PLC achieves interoperability, scalability, and safety without interfering with control logic. Its implementation and validation in a real automotive factory demonstrate how architectural design choices aligned with industrial constraints can facilitate the large-scale adoption of data-driven sensor network applications in Industry 4.0.

### 2.2. Communication Performance, Coverage, and Analytical Modeling

In the paper “VistaScan: Optimizing Internet-Wide Scanning Through Visibility-Aware Distributed Task Allocation” (Contribution 1), Hu et al. address efficient Internet-scale network measurement for security and monitoring. The authors highlight visibility heterogeneity across networks as a key limitation of both centralized and naive distributed scanning, leading to redundant probing and incomplete coverage.

To address this issue, VistaScan leverages the principle of visibility consistency, assuming that IP addresses within the same CIDR block share similar visibility patterns. Using anchor-based probing, the system constructs a lightweight visibility matrix and applies a load-aware task allocation strategy that assigns scanning tasks to suitable nodes while filtering unreachable targets. Experimental results show that VistaScan achieves high coverage with significantly reduced probing overhead and scan time, while generalizing across services. This work demonstrates how visibility-aware modeling and coordinated task allocation enable scalable and efficient Internet-wide scanning.

Silva et al., in the paper “Radio Coverage Assessment and Indoor Communication Enhancement in Hospitals: A Case Study at CHUCB” (Contribution 3), investigate the performance of cellular communication technologies in a complex indoor healthcare environment. Through a measurement campaign conducted at the Cova da Beira University Hospital Center, the authors evaluate signal strength and quality for multiple technologies, including 5G NR, LTE, NB-IoT, and UMTS, using professional-grade spectrum analysis equipment.

The study identifies specific indoor locations with insufficient coverage and analyzes the performance differences among mobile network operators. Based on these findings, the authors propose the targeted deployment of femtocells as a scalable and replicable solution to enhance indoor connectivity. Beyond coverage improvement, the work highlights the broader implications for healthcare sensor networks, including reliable telemonitoring, real-time tracking of equipment and patients, and secure data transmission, particularly in clinical areas and resource-constrained settings.

In Contribution 10, Rodriguez-Gomez et al. revisit the classical CSMA non-persistent medium access protocol from a perspective relevant to modern sensor networks. While most analytical models assume an infinite node population, the authors demonstrate that this assumption leads to significant inaccuracies in scenarios with a limited number of active nodes and low traffic intensity.

The authors derive a closed-form expression for system throughput that explicitly accounts for a finite number of nodes, complemented by a computationally efficient approximation. Through numerical evaluation and simulation-based validation, the study reveals how throughput, delay, and energy consumption characteristics vary with node population size. These results provide important insights for the design and analysis of contention-based MAC protocols in practical sensor network deployments where the infinite-population assumption no longer holds.

### 2.3. Intelligent Processing and Learning-Driven Optimization in Sensor Networks

In the paper “EdgeVidCap: A Channel-Spatial Dual-Branch Lightweight Video Captioning Model for IoT Edge Cameras” (Contribution 4), Guo et al. address the challenge of deploying advanced video understanding algorithms on resource-constrained IoT edge devices. The authors note that conventional video captioning models are computationally intensive and unsuitable for real-time processing on embedded platforms.

To address this issue, the paper introduces a lightweight architecture that integrates channel attention mechanisms with state-space models through a novel Synergetic Attention State Mamba module. An adaptive attention-guided LSTM decoder further enhances caption generation by dynamically weighting visual features. Experimental evaluations on benchmark datasets demonstrate that EdgeVidCap achieves competitive captioning accuracy while significantly reducing model complexity and computational requirements, enabling practical deployment on intelligent edge cameras.

The paper “DRL-Driven Intelligent SFC Deployment in MEC Workload for Dynamic IoT Networks” (Contribution 5), by Ros, Ryoo, and Kim, proposes a learning-based framework for resource orchestration in IoT systems integrated with multi-access edge computing. The authors address the challenges posed by fluctuating workloads, limited edge resources, and stringent quality-of-service requirements in dynamic IoT environments.

The proposed framework leverages deep reinforcement learning (DRL), specifically a Deep Q-Network, to jointly optimize task offloading decisions, service function chaining, virtual network function placement, and routing. Simulation results demonstrate improvements in latency, energy consumption, packet delivery ratio, and throughput compared to baseline approaches. This contribution highlights the effectiveness of DRL in managing complex, multi-dimensional optimization problems in large-scale sensor network ecosystems.

In Contribution 9, Eriş, Gül, and Bölük focus on sustainable communication in underwater acoustic sensor networks, where battery replacement is often impractical. The authors consider a realistic stochastic model for piezoelectric energy harvesting and the spatio-temporal variability of underwater ambient resources.

They propose a distributed multi-agent reinforcement learning–based TDMA scheduling scheme that allows sensor nodes to adapt their transmission slots based on available harvested energy autonomously. Simulation results demonstrate improvements in throughput and network lifetime metrics, including the first-, half-, and last-node death times. The work illustrates how learning-enabled MAC protocols can effectively exploit ambient energy sources and enhance the long-term sustainability of underwater sensor networks.

### 2.4. Energy Efficiency, Embedded Platforms, and Security in Sensor Networks

The paper “A Cluster Head Selection Algorithm for Extending Last Node Lifetime in Wireless Sensor Networks” (Contribution 6) revisits the problem of energy-efficient clustering in wireless sensor networks. Unlike traditional approaches that aim to balance energy consumption across nodes, the authors formulate network lifetime as a function of node energy depletion and analytically derive a cluster head selection rule that maximizes the time until the last node dies.

The proposed algorithm prioritizes nodes with high transmission probabilities and low initial energy levels for cluster head selection and operates using distributed per-cluster computation. Its effectiveness is validated through experiments on a real LoRaWAN-based sensor network prototype, demonstrating significant lifetime improvements over state-of-the-art methods. This contribution emphasizes the importance of analytically grounded, application-aware algorithms validated in real-world deployments.

In Contribution 8, Vandervelden et al. provide a comprehensive survey and comparative analysis of Rust-based operating systems and frameworks for sensor nodes. Motivated by Rust’s memory safety guarantees, the authors examine platforms such as Tock, Hubris, RTIC, and Embassy, focusing on scheduling, inter-process communication, application isolation, and networking support.

The paper also presents a performance evaluation of scheduling latency and memory footprint, highlighting trade-offs between functionality and resource usage. By contrasting different design philosophies, this contribution offers valuable guidance for developers seeking secure and efficient software platforms for embedded sensor network devices.

Lee et al., in the paper “A Programmable Crypto-Processor for National Institute of Standards and Technology Post-Quantum Cryptography Standardization Based on the RISC-V Architecture” (Contribution 11), address emerging security challenges posed by quantum computing. The authors propose a programmable crypto-processor that extends the RISC-V instruction set to accelerate post-quantum cryptographic algorithms currently under NIST standardization.

The proposed architecture reduces code size, instruction count, and execution cycles for key cryptographic operations while maintaining flexibility to support multiple algorithms. Implemented as a coprocessor, the design enables efficient and secure cryptographic processing in embedded systems. This contribution demonstrates how hardware–software co-design can play a critical role in ensuring long-term security for future sensor network deployments.
